# Natural History of NAFLD Diagnosed in Childhood: A Single-Center Study

**DOI:** 10.3390/children4050034

**Published:** 2017-05-03

**Authors:** Catherine E. Cioffi, Jean A. Welsh, Rebecca L. Cleeton, Shelley A. Caltharp, Rene Romero, Mark L. Wulkan, Juna V. Konomi, Jennifer K. Frediani, Miriam B. Vos

**Affiliations:** 1 Laney Graduate School, Emory University, Atlanta, GA 30322, USA; catherine.cioffi@emory.edu; 2 Division of Gastroenterology, Hepatology and Nutrition, Department of Pediatrics, School of Medicine, Emory University, Atlanta, GA 30322, USA; jean.a.welsh@emory.edu (J.A.W.); rebecca.lebo.cleeton@emory.edu (R.L.C.); rromero@emory.edu (R.R.); junakonomi@emory.edu (J.V.K.); jfredia@emory.edu (J.K.F.); 3 Children’s Healthcare of Atlanta, Atlanta, GA 30322, USA; 4 Division of Pediatric Surgery, Department of Surgery, School of Medicine, Emory University, Atlanta, GA 30322, USA; mwulkan@emory.edu

**Keywords:** adolescence, BMI, hepatic, metabolic syndrome, longitudinal

## Abstract

Little is known regarding the subsequent course of non-alcoholic fatty liver disease (NAFLD) diagnosed in childhood. The objectives of this single-center study were to gather data on long-term health outcomes and to assess the feasibility of contacting former pediatric patients. In a large pediatric medical center, electronic records were searched to initially identify 162 former patients who had a liver biopsy between 2000 and 2010. Of these, 44 subjects met the criteria for age at follow-up (≥18 year) and biopsy-proven NAFLD, and were recruited via postal and electronic mail. Participants were invited to complete a brief telephone survey on current health status. Supplemental data was also obtained from pediatric medical charts of all subjects. At NAFLD diagnosis, 18% of subjects had diabetes, 91% were obese, 61% had NASH, and 56% had fibrosis on biopsy. At follow-up, 10 subjects (23%) responded to the survey. Based on the survey and chart review, after a mean follow-up of 4.5 years, 5 additional subjects developed diabetes for a period prevalence of 30%, and most subjects (78%) remained obese at last follow-up. Additional prospective studies are needed to fully describe the longitudinal risks associated with pediatric NAFLD, and will require multi-dimensional strategies to successfully recruit former patients.

## 1. Introduction

The prevalence of pediatric non-alcoholic fatty liver disease (NAFLD) has increased considerably in parallel with trends in childhood overweight and obesity [1,2,3,4]. In addition to obesity, NAFLD is strongly associated with metabolic syndrome [5]. Cross-sectional and short-term cohort studies (<3 years) have shown that children with NAFLD have higher fasting insulin and blood glucose levels, and increased risk of dyslipidemia, hypertension, and inflammatory markers compared to non-NAFLD children [6,7,8,9]. In adult NAFLD patients, natural history studies have found not only hepatic outcomes of cirrhosis and hepatocellular carcinoma, but also increased incidence of type 2 diabetes [10], and higher rates of morbidity and mortality from cardiovascular disease (CVD) [11]. However, few long-term natural history studies exist in children and adolescents with NAFLD.

One of the biggest challenges in pediatric natural history studies involves the transition of care to adult medical providers, as patients “age out.” There are also many barriers to locating former pediatric patients once they become young adults, including increased mobility among young adults due to educational and employment opportunities, marriage and other life changes, greater use of cell phones, and limited access to health insurance [12]. Feldstein et al. overcame this problem by collecting information retrospectively on 66 children with NAFLD over time periods up to 20 years, and reported a 13-fold increased rate of mortality compared to the general population [13]. This study was limited, however, by having only 43% of the sample diagnosed by liver biopsy, which is the preferred method of confirming the degree of steatosis and other histopathological features. A-Kader et al. also collected follow-up data on 18 children with biopsy-proven NAFLD over a mean period of 28 months, and found some patients had progression of fibrosis on repeat biopsy, while other patients had improvements in fibrosis over time, especially with weight loss [14]. However, their follow-up did not report on metabolic co-morbidities. Thus, confirmatory longitudinal studies examining the health risks and co-morbidities of NAFLD for children as they continue into adulthood are needed.

The objectives of this pilot study were to assess health outcomes among individuals diagnosed with NAFLD as children, particularly mortality and type 2 diabetes, and to test methodologies for a larger-scale longitudinal pediatric NAFLD follow-up study.

## 2. Materials and Methods

### 2.1. Subjects

The study methods were approved by the Emory University (Atlanta, GA, USA) and Children’s Healthcare of Atlanta institutional review boards (IRBs). The electronic pathology database of Children’s Healthcare of Atlanta (CHOA) was searched to identify an initial list of eligible participants who had a liver biopsy between 2000 and 2010. The search terms used were “macrovesicular steatosis” OR “fatty change liver” OR “steatohepatitis” OR “NAFLD” OR (liver AND steatosis) OR “fatty liver” OR (steatosis AND liver). A Health Insurance Portability and Accountability Act (HIPAA) waiver was granted to review the medical records of eligible patients identified in the pathology search. This review was performed by two researchers separately and checked for consistency. Individuals were excluded if at time of review they were <18 years of age, if their liver biopsy was negative for steatosis, or if the steatosis noted on biopsy was caused by a separate steatogenic condition, such as viral liver disease, Wilson’s disease, autoimmune hepatitis, mitochondrial disorders, or steroids or other medications known to cause steatosis. In the pathology database search, a subgroup of patients had a liver biopsy performed concurrent to bariatric surgery. Since the subjects were adolescents at the time of their bariatric surgery, they were placed under an investigational device exemption (IDE) according to the U.S. Food & Drug Administration (FDA). These patients were included in the study if their liver biopsy indicated at least minimal steatosis and inflammation or fibrosis, with the consideration that all patients in this group underwent a several week long very low calorie regimen to decrease hepatic size as preparation for the surgery [15], which has been shown in prior to studies to also acutely decrease the amount of intrahepatic steatosis [16].

### 2.2. Survey Implementation

Patient contact information (mailing address, phone number, and email) was obtained using the LexisNexis search engine. All eligible subjects were mailed a cover letter introducing the study and eligibility, the survey, an informed consent information sheet, and a gift card. One to two weeks following the initial mailing, each patient was contacted by telephone by the study team to document verbal consent, obtain responses to the brief survey, and confirm their mailing address to send compensation and resources on NAFLD treatment (if interest was indicated). Compensation was provided in phases: first, five dollars was mailed with the initial cover letter to incentivize response, and an additional twenty dollars was mailed after completion of the brief survey. Each participant was called at most three times on different days and times, and voicemails were left, if possible. Electronic mail was also sent to each participant if the phone calls were unsuccessful. After all measures were taken, subjects were classified as responder, deceased, non-responder with no verbal contact (the study team could not reach the patient or his/her family), or non-responder with verbal contact (the study team spoke with the patient’s family, but not the patient).

### 2.3. Survey Design & Data Collection

The pilot survey was designed to be brief to reduce respondent burden. It included seven questions to assess medical-care seeking behaviors; health outcomes (Type 2 diabetes, high blood pressure, high cholesterol, high triglycerides, or cardiovascular); current weight status (height and weight); and willingness to participate in additional research activities (Table S1).

### 2.4. Review of Medical Record

Each patient’s medical record was reviewed for anthropometric data, including height and weight, and laboratory values including liver enzymes (alanine aminotransferase. (ALT) and aspartate aminotransferase (AST), gamma-glutamyltransferase (GGT)), lipids (total cholesterol, triglycerides, and high-density lipoprotein (HDL) cholesterol), and glucose levels at the time of biopsy diagnosis. Body mass index (BMI) was calculated as weight (kg) divided by height-squared (m^2^). Age and sex-specific BMI z-scores and percentiles were calculated using the 2000 Centers for Disease Control and Prevention (CDC) growth charts. Obesity at baseline was classified by BMI at or greater than the 95^th^ percentile. Histological features of liver biopsies were also recorded, including degree of steatosis (mild-moderate or severe), presence of fibrosis (yes/no), and non-alcoholic steatohepatitis (NASH) (yes/no). Each pediatric chart was also reviewed for comorbidities, including type 2 diabetes (fasting glucose ≥ 126 mg/dL or 2-h glucose ≥ 200 mg/dL), hypertension, and dyslipidemia. The date of diagnosis was determined by first mention in the medical record. Charts were also reviewed for prediabetes, based on impaired fasting glucose (fasting plasma glucose between 100 and 125 mg/dL), impaired glucose tolerance (2-h glucose between 140 and 199 mg/dL), or both [17]. Among survey non-responders, the last height and weight in the medical chart and the date of these measurements were also recorded to determine adult BMI and age at last follow-up.

### 2.5. Data Analysis

Descriptive statistics were reported as mean ± standard deviation for continuous and counts and frequencies (%) for categorical variables. Two-sample independent *t*-tests or Fisher’s exact tests compared characteristics of the subgroups; i.e., NAFLD subjects with vs. without bariatric surgery. Survival analysis using Kaplan-Meier methods assessed time to diabetes diagnosis, with time starting from birth to assess lifetime survival risk, or from liver biopsy to assess survival risk after NAFLD diagnosis. The “event” was defined as diabetes only, or diabetes and prediabetes diagnosis. Subjects were censored at last encounter in the medical record or date of survey response.

Longitudinal linear mixed-effects models with unstructured covariance structure were used to assess change in BMI from baseline to last follow-up, as these models allow for random variation within and between subjects, while also accounting for measurements at irregular time points of observation. The model was fit with “subject” as a random effect, “age” (in years) as a random and fixed effect, and a random intercept and slope. Parameter values were calculated using maximum likelihood estimation. An interaction model with “age × subgroup”, and their main effects, was also fit to assess heterogeneity in BMI trends by subgroup, as we hypothesized that weight loss surgery may have impacted BMI at baseline and change over time. Finally, logistic regression models tested if age, follow-up time, and/or gender were associated with odds of responding to the survey. SAS Statistical Software version 9.4 (SAS Institute, Cary, NC, USA) was used for the analysis. An alpha-level of 0.05 was established.

## 3. Results

**Figure 1:**
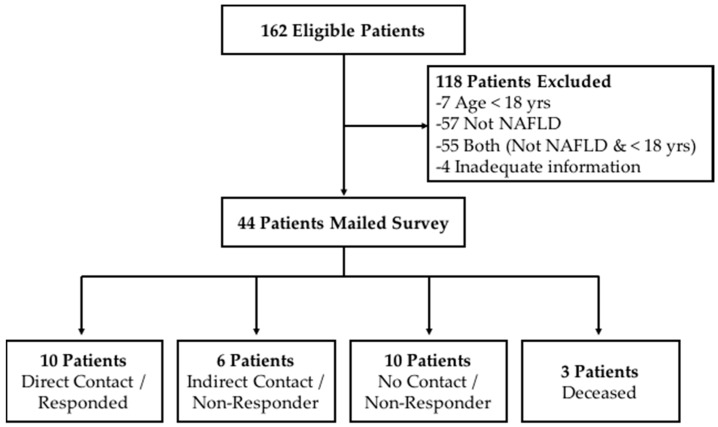
Flow diagram of eligible subjects throughout the study.

Liver biopsies from a total of 162 pediatric patients were reviewed and 131 were excluded because their age at the time of this study was not 18 years or older and/or they had a condition other than NAFLD, or there was inadequate information to determine a NAFLD diagnosis (Figure 1). The final sample included 44 eligible young adults with biopsy-proven NAFLD, defined as having at least 5% steatosis, of which 14 were diagnosed based on liver biopsy performed at the time of adjustable gastric band surgery, a form of bariatric surgery for obesity treatment. 

Table 1 summarizes the characteristics of the sample overall, and by subgroup (i.e., NAFLD-only versus bariatric surgery patients) as we suspected the two groups may have different baseline characteristics. NAFLD-only subjects were predominantly male, non-Hispanic White or Hispanic, and diagnosed at a mean age of 14.0 ± 2.8 years. Most of these subjects (60%) had mild-moderate steatosis, and more than 50% had fibrosis and 80% had steatohepatitis on biopsy. Two of the NAFLD-only subjects were noted to have cirrhotic changes on their initial biopsy. In contrast, the bariatric surgery subjects tended to be female, Caucasian or African American, and diagnosed at a mean age of 16.4 ± 1.2 years. The majority had mild-moderate steatosis (80%), and signs of fibrosis (57%) prior to surgery on liver biopsy, but a smaller percentage had steatohepatitis (21%). In the full sample, most subjects (91%) were obese at diagnosis, however the mean BMI was significantly greater in subjects who had bariatric surgery compared to those who did not (*p* < 0.01). Mean laboratory values at the time of their liver biopsy diagnosis can be found in Table S2.

### 3.1. Survey Response Rates

Ten of 44 subjects responded to the survey, achieving a 22.7% response rate. All respondents were recruited by telephone calls. There were no respondents who we made verbal contact with but who refused to participate in the study. We spoke directly to the family members (i.e., parents or guardians) of six eligible subjects while conducting the telephone calls, but did not receive a return phone call from the participant themselves so they were not included the survey. The remaining eligible subjects were either non-responders (no direct or indirect verbal contact) or deceased.

### 3.2. Long-Term Follow-Up

The average time of follow-up was 4.5 ± 2.9 years, or a total of 143.7 person years, from liver biopsy to last recorded height and weight in the medical record (for survey non-respondents), or to survey completion (Table 2). Twelve subjects were censored immediately after diagnosis because no further notes were available in the pediatric medical record and they did not respond to the survey. The mean age at follow-up for the sample after excluding these 12 subjects was 19.0 ± 4.1 years. Among the 10 survey respondents, the mean age at follow-up was 22.8 ± 3.0 years.

**Figure 2:**
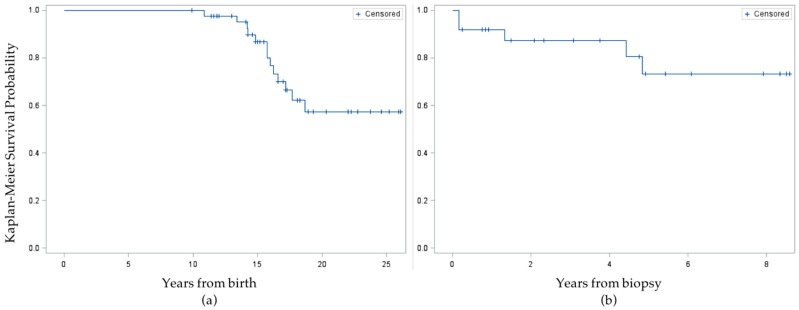
Kaplan-Meier survival curves for time to diabetes diagnosis. (**a**) Time to event starting from birth for the full sample of subjects; (**b**) Time to event starting at liver biopsy, excluding *n* = 12 subjects who were censored at time of biopsy.

None of the 10 survey respondents reported ever being told by a healthcare professional that they had high blood pressure, dyslipidemia, or cardiovascular disease, but one respondent reported being told they have/had type 2 diabetes. When supplemented with information from the medical chart review of all 44 eligible subjects, an additional 12 had been diagnosed with type 2 diabetes ever, including one survey respondent who misreported not having a history of diabetes on the telephone survey, for a total of 13 cases. Eight cases were diagnosed prior to or concurrent with liver biopsy, for a baseline prevalence of 18.2%. After diagnosis of NAFLD, the incidence rate was 5/104.1 person years (48 per thousand) among all other subjects. In Kaplan-Meier survival analysis of time to diabetes diagnosis, the mean survival time was 17.5 ± 0.3 years from birth or 4.3 ± 0.3 years from liver biopsy (Figure 2). When the event was time to diabetes *or* prediabetes diagnosis, the mean survival time from birth was similar, at 17.2 ± 0.4 years, and survival time from liver biopsy was unchanged.

The overall mean BMI at follow-up was 39.0 kg/m^2^, but subjects who had undergone Lap Band surgery remained at a significantly higher mean BMI than those who did not (*p* < 0.01; Table 2). In the crude linear mixed effects model (i.e., unconditional growth model) of change in BMI from baseline to follow-up age, the random intercept was significant (*p* < 0.01), suggesting significant between-person variation at baseline, but the slope term (age) was not significant (*p* = 0.30) suggesting no significant variation in the average rate of change in BMI as age increased (Table 3).

**Figure 3:**
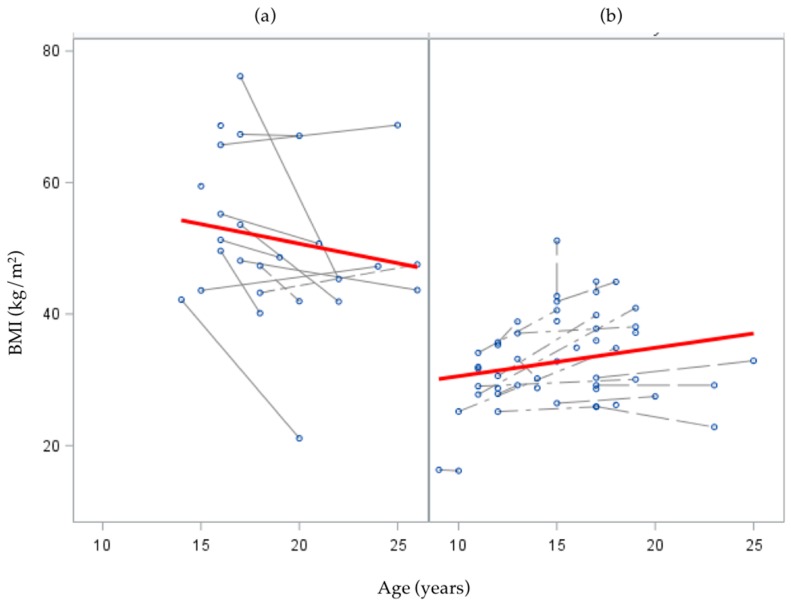
Change in BMI (kg/m^2^) from baseline to follow-up for each subject (dotted lines) and overall (solid line), stratified by subgroup: (**a**) Bariatric surgery subjects or (**b**) NAFLD-only subjects. Excludes *n* = 12 subjects who were lost-to-follow-up after biopsy diagnosis.

In the interaction model with subgroup × age interaction, and their main effects, subgroup was a significant predictor of initial status ($\beta_{subgroup\;}$ = 23.4, *p* < 0.01); such that the mean BMI for the bariatric surgery group was significantly greater than that of the NAFLD-only group. The subgroup × age interaction was also significant ($\beta_{subgroup\;\times \;age\;}$ = −1.29, *p* < 0.01), suggesting the rate of change in BMI as age increased was different for subgroups. Specifically, change in BMI over follow-up was negative (decreasing) over time for the bariatric surgery group, while NAFLD-only subjects had a positive (increasing) trend in BMI change over time, though this latter trend did not reach statistical significance (*p* = 0.08; Figure 3).

Regarding liver disease related outcomes, three of 44 subjects had one repeat biopsy in their pediatric medical chart (mean follow-up: 4.2 ± 2.5 years after their first biopsy. All 3 subjects had steatohepatitis (NASH) at baseline. Two of the 3 subjects experienced progression in the degree of steatosis and fibrosis from the first biopsy, while the third subject had stable NASH and unchanged fibrosis compared to his/her initial biopsy. No subjects were found to have had a liver transplant, but three of the 44 NAFLD subjects (7%) were deceased at follow-up. This was recorded in the medical chart for one participant, who suffered an immune disorder in addition to being diagnosed with NAFLD. The two other subjects were confirmed deceased after speaking with their family members during telephone call recruitment. Both had a past surgical history of adjustable gastric band placement and by parent report died from non-liver related causes.

### 3.3. Feasibility of Future Research Activities

Of the 10 subjects who responded to the survey, all consented to participate during the initial contact period over the telephone. We did not receive additional responses by expanding the recruitment to include an email contact. There were no differences in odds of responding by current age or gender, but the amount of follow-up time since biopsy was a marginally significant predictor. Specifically, as follow-up time from diagnosis increased, the odds of responding to the survey decreased (*p* = 0.05). The majority (8 of 10) of respondents indicated that they would be willing to complete an additional longer survey for compensation. Among these respondents, all preferred receiving the survey by email and completing it online. Additionally, 7 of 10 respondents said that they would be interested in being contacted for future research opportunities or studies.

## 4. Discussion

This study collected follow-up information on health outcomes of former pediatric NAFLD patients who are now young adults (between ages 18 and 30 years old). In general, research targeting this age group has been limited and often overlooked, despite this transition period representing a critical time in the life span [18,19] Young adulthood is marked by independence and initiation of adult behaviors, and can be a significant and challenging time for health and health care access. A recent review of young adult health over the past decade found that, although there have been improvements in certain health outcomes, there have also been negative trends, including increased rates of obesity, diabetes, and heart disease [20].

In our survey of former pediatric NAFLD patients, the majority of respondents self-reported a current height and weight at follow-up that would be categorized according to World Health Organization as either overweight or obese. This high prevalence of obesity at follow-up was also seen in the full sample after adding supplementary information from the medical record for non-responders. There was, however, heterogeneity in the sample. Specifically, mean BMI decreased among the bariatric patients as age increased, though this group remained obese at follow-up, which aligns with findings from Inge et al. on long-term (3-year) outcomes of bariatric surgery in adolescence [21]. Conversely, among NAFLD-only patients, BMI increased with age, which is concerning from a long-term health perspective given the strong associations between obesity and chronic disease among adults.

We found a period prevalence of 29.6% for type II diabetes, which included 8 of 44 patients who had diabetes at baseline. This is higher than the prevalence previously published in children but close to the rates published in adults with NAFLD [22,23,24], and assumes that none of the cases of type 2 diabetes resolved after last follow-up in the pediatric medical record. On the other hand, it may also be an underestimate as we were limited by a low response rate, loss to follow-up, and access to only pediatric healthcare records. This also did not include the patients with prediabetes, and who may have progressed to type 2 diabetes.

Although none of the respondents reported ever having been told that they had metabolic syndrome components such as dyslipidemia or hypertension, supplementary information from the medical record indicated that at least a portion of subjects had been, at one point, diagnosed with one or more of these conditions [25]. Children with NAFLD often present with metabolic syndrome components, such as higher concentrations of cholesterol and triglyceride, and lower concentrations of HDL [26,27,28]. It is possible that respondents forgot if they had been told that they had one of these conditions when they were younger or had misunderstood the survey question, or that the condition might have resolved. As evidenced by this discrepancy, future research will require participants to undergo testing as self-report appears unreliable for these conditions.

This study also contributed information on response rates among the target population, which will be useful for the design of future studies. Because we started with a small pool of eligible participants (*n* = 44), the absolute sample size of respondents we achieved was also small (*n* = 10), corresponding to a response rate of 23%. However, based on the literature, this rate is within the response range achieved in similar studies, which have been reported as high as 48.2%, or as low as 17.5% in the 2007 Survey of Adult Transition and Health (SATH) [12,29,30]. Potential reasons for the low response rate in our study and others are the increased mobility during this time in the lifespan, characterized by transitions to college and/or new employment, greater use of cell phones, and lower likelihood of having health insurance or access to healthcare.

One limitation was the study’s reliance on self-report data for the survey, which allows for the possibility of recall and/or response bias, whereby subjects may have difficulty remembering the correct information and/or may have responded in a way that would be perceived as more desirable, respectively. For example, patients may have underestimated their weight or failed to report a history of one of the medical conditions to appeal more “acceptable” to the research team. Additionally, the survey administered was brief to reduce respondent burden, but there remains unanswered questions about the respondents’ health and lifestyle characteristics due to its limited number of questions. The use of available medical records to abstract information at baseline and over follow-up was also a limitation, as we were only able to report on clinical data that was recorded in the sample’s pediatric chart. We did not have access to additional outcomes for all subjects, for example degree of insulin resistance or progression of liver disease histopathological outcomes. Having access to adult health records would also have been beneficial, as this would have provided insight into access to care and additional long-term health outcomes after pediatric discharge. Finally, although alcohol use was assessed in some subjects at the time of NAFLD diagnosis, it was not consistently reported.

Strengths of the study include the systematic search methods used to identify eligible patients. We included a wide timeframe of eligible biopsies, spanning an entire decade, and collaborated with the pathology department to ensure that the search terms were general enough to include all possible NAFLD cases, which were then narrowed down by two reviewers to ensure the accuracy and validity of each diagnosis. Furthermore, by only including patients with biopsy-proven NAFLD, which is the gold standard for NAFLD diagnosis, we are more confident in the accuracy of their diagnosis. We also utilized various recruitment strategies, within the allowance of our Institutional Review Board, to appeal to different contact preferences of the eligible participants, and combined the resources available in the pediatric electronic medical record and LexisNexis search engine to collect the most up-to-date contact information possible.

In summary, this study highlights the importance and challenges of longitudinal studies in the United States of former pediatric NAFLD patients during and after the transition into adulthood. Our data support that children with NAFLD are at very high risk of type 2 diabetes as young adults. With continual improvements and continuity in the electronic medical record systems, it may become easier to follow patients through this transition from pediatric to adult healthcare. Given the likelihood of low response rates, collaborative efforts with multiple sites and health systems may help to achieve a more adequate sample size for pediatric NAFLD natural history studies, and will allow us to make more reliable and accurate estimates regarding the long-term prognosis of the disease. While the data were limited, the high rate of type II diabetes (30%) and persistence of obesity among former pediatric NAFLD patients from diagnosis to follow-up into young adulthood are concerning and should be evaluated in larger studies.

## Figures and Tables

**Table 1:** Demographics of sample at time of biopsy.

	Full Sample *N* = 44	Subgroup	
		NAFLD-Only *n* = 30	Bariatric Surgery *n* = 14
Age (years)	14.8 ± 2.6	14.0 ± 2.8	16.4 ± 1.2
BMI (kg/m^2^)	39.4 ± 13.5	31.9 ± 6.3	55.1 ± 10.8
BMI z-score	2.3 ± 0.6	2.1 ± 0.6	2.8 ± 0.3
Obesity^1^	39 (90.7%)	25 (86.2%)	14 (100%)
Male	23 (52.3%)	20 (66.7%)	3 (21.4%)
**Race/ethnicity**			
Non-Hispanic Black	6 (13.6%)	1 (3.3%)	5 (35.7%)
Hispanic/Latino	13 (29.6%)	12 (40.0%)	1 (7.1%))
Non-Hispanic White	21 (47.7%)	13 (43.3%)	8 (57.1%)
Asian	4 (9.1%)	4 (13.3%)	–
**Steatosis score**			
Mild-Moderate (5–66%)	29 (65.9%)	18 (60.0%)	11 (78.8%)
Severe (>66%)	9 (20.5%)	6 (20.0%)	3 (21.4%)
Not specified	6 (13.6%)	6 (20.0%)	–
**Histological Features**			
Fibrosis (y/n)	25 (56.8%)	17 (56.7%)	8 (57.1%)
Steatohepatitis (y/n)	27 (61.4%)	24 (80.0%)	3 (21.4%)

NAFLD: non-alcoholic fatty liver; BMI: body mass index. Mean ± SD for continuous and *n* (%) for categorical variables; ^1^ Obesity was defined according to sex- and age-specific BMI ≥ 95th percentile according to the 2000 Centers for Disease Control and Prevention (CDC) growth charts.

**Table 2:** Health outcomes in the overall sample and by subgroup

Survey Outcomes ^2^				
	Full Sample	Subgroup		
		NAFLD-Only	Bariatric Surgery	
**Respondents**	10/44 (22.7%)	6/30 (20.0%)	4/14 (28.5%)	*p*-value ^1^
**Follow-up time (years)**	7.7 ± 2.9	7.2 ± 1.5	8.4 ± 0.3	0.104
**Age at F/U (years)**	22.8 ± 2.9	21.3 ± 2.9	25.1 ± 1.1	0.037
**BMI at F/U (kg/m^2^)**	39.5 ± 2.9	31.3 ± 5.3	51.8 ± 11.4	0.005
**Obese ^3^**	8 (80%)	4 (67%)	4 (100%)	0.467
**Type 2 Diabetes**	1 (10%)	1 (7%)	0 (0%)	0.389
**Medical Chart Outcomes ^3^**				
		**Subgroup**		
	**Full Sample**	**NAFLD-Only**	**Bariatric Surgery**	
	***n* = 32**	***n* = 20**	***n* = 12**	***p*-Value ^1^**
**Follow-up time (years)**	4.5 ± 2.9	3.9 ± 3.1	5.4 ± 2.5	0.164
**Age at F/U (years)**	19.0 ± 4.1	17.3 ± 3.9	21.9 ± 2.6	0.001
**BMI at F/U (kg/m^2^)**	39.0 ± 11.7	34.2 ± 8.3	47.0 ± 12.3	0.001
**Obese**	25 (78%)	14 (70%)	11 (92%)	0.212
**Type 2 Diabetes**	13 (30%)	9 (30%)	4 (29%)	0.923
**Hypertension**	10 (23%)	7 (23%)	3 (22%)	0.888
**High Cholesterol**	16 (36%)	15 (50%)	1 (7%)	0.007
**High Triglycerides**	21 (48%)	19 (63%)	2 (14%)	0.003
**Low HDL-cholesterol**	17 (39%)	16 (53%)	1 (7%)	0.004

^1^ *p*-value calculated using an independent *t*-test or Fisher’s exact test to compare means or proportions, respectively, for NAFLD-only vs. bariatric surgery subjects; ^2^ Survey outcomes not reported were self-reported history of elevated cholesterol, elevated triglycerides, hypertension, and cardiovascular disease because no respondents indicated having these conditions; ^3^ Denominator for percentages under medical chart outcomes is indicated under the “eligible subjects” row, which excludes *n* = 12 subjects with no follow-up after biopsy; i.e., time of censoring was time of biopsy.

**Table 3:** Estimates from linear mixed effect models of longitudinal BMI.

Model	Exposure	β	SE	*p*
**A (Crude)**	Intercept	38.6	2.0	<0.001
	Age ^1^	0.3	0.3	0.336
**B (Adjusted)**	Intercept	32.2	1.5	<0.001
	Age	0.5	0.3	0.078
	Bariatric Subgroup	23.4	3.1	<0.001
	Subgroup × Age	−1.3	0.5	0.007

^1^ Age was centered at 14 years old, the mean age at baseline, for interpretation of the intercept.

## Supplementary Materials

The following are available online at http://www.mdpi.com/2227-9067/4/5/34/s1, Table S1: Questions administered in the pilot survey, Table S2: Laboratory values among eligible subjects at biopsy diagnosis.
