# Supplementary material for: Natural History of NAFLD Diagnosed in Childhood: A Single-Center Study
Source: Children (Basel). 2017 May 3;4(5):34. doi: 10.3390/children4050034 (PMC5447992; doi:10.3390/children4050034)
Supplement: Supplementary file 1 [file children-04-00034-s001.pdf]

Supplementary Materials: ***Natural history of NAFLD diagnosed in childhood: A Single-Center Study***

Table S1: Questions administered in the brief pilot survey

1. Do you currently see a doctor for reasons relating to your liver disease? (Yes/No)
    a. If Yes, How frequently?
2. Please check below if a doctor or other healthcare provider has told you that you have any of the following conditions:
    a. Type II Diabetes
    b. Elevated Blood Pressure
    c. Elevated Total Cholesterol
    d. Elevated Triglycerides
    e. Cardiovascular Disease
3. What is your current height and weight?
4. Would you be willing to complete a longer survey about liver disease? If you choose yes, you will receive additional compensation for the time that would be required (Yes/No)
    a. If yes, please indicate below if you prefer to be reached by telephone to answer the additional survey questions, or if you prefer to complete the survey online.
5. Are you interested in receiving more information about healthcare providers, treatment options, and other resources about fatty liver disease? (Yes/No)
6. Would you be interested in participating in additional future research studies conducted by our research team at Children's Healthcare of Atlanta relating to liver disease? (Yes/No)

Table S2: Laboratory values among eligible subjects at biopsy diagnosis.

**Laboratory Values:**	**N[1]**	**Mean ± SD**	**Range (Min – Max)**	**N (%) Abnormal**	**Normal Range[2]**
ALT (U/L)	33	143.8 ± 133.0	27.0 – 686	31 (70.5%)	< 26 U/L
AST (U/L)	33	98.7 ± 77.5	19.0 – 391	32 (97.0%)	< 31 U/L
AST/ALT Ratio	33	0.8 ± 0.4	0.2 – 2.1	6 (18.2%)	< 1:1
GGT (U/L)	25	86.7 ± 92.2	22.0 – 411.0	19 (76.0%)	< 31 U/L
Triglyceride (mg/dL)	19	200.8 ± 81.3	70.0 – 351.0	13 (68.4%)	< 150 mg/dL
Total Cholesterol (mg/dL)	20	186.2 ± 46.9	92.0 – 291.0	8 (40.0%)	< 200 mg/dL
HDL-Cholesterol (mg/dL)	17	36.4 ± 9.0	18.0 – 55.0	12 (70.6%)	> 35 mg/dL
LDL-Cholesterol (mg/dL)	14	126.4 ± 28.2	69.0 – 163.0	8 (57.1%)	< 130 mg/dL
Glucose (mg/dL)	35	107.0 ± 33.6	76.0 – 223.0	6 (17.1%)	< 126 mg/dL

[1] N refers to number of subjects with non-missing values for the clinical laboratory value.

[2] Normal ranges based are specified for children and adolescents approximately age 12-19 yrs old.
